# Enhanced light trapping in solar cells using *snow globe coating*

**DOI:** 10.1002/pip.2240

**Published:** 2012-05-15

**Authors:** Angelika Basch, Fiona Beck, Thomas Söderström, Sergey Varlamov, Kylie R Catchpole

**Affiliations:** 1 Centre for Sustainable Energy Systems, The Australian National UniversityCanberra, ACT, 0200, Australia; 2 Institute of Physics, University of GrazUniversitätsplatz 5, 8010, Graz, Austria; 3 ICFO - The Institute of Photonic SciencesBarcelona, Spain; 4 ARC Centre of Excellence for Advanced Silicon Photovoltaics and Photonics, University of NSWSydney, NSW, 2052, Australia

**Keywords:** light trapping, semiconductors, dielectric materials, zeta-potential, thin films, refractive index

## Abstract

A novel method, *snow globe coating*, is found to show significant enhancement of the short circuit current *J_SC_* (35%) when applied as a scattering back reflector for polycrystalline silicon thin-film solar cells. The coating is formed from high refractive index titania particles without containing binder and gives close to 100% reflectance for wavelengths above 400 nm. Snow globe coating is a physicochemical coating method executable in pH neutral media. The mild conditions of this process make this method applicable to many different types of solar cells. Copyright © 2012 John Wiley & Sons, Ltd.

## 1. INTRODUCTION

Photovoltaics is a well-developed technology, but needs to be cheaper to create sustainable energy sources that can compete with conventional fossil fuels [1]. Solar cells based on silicon wafers are by far the most dominant technology, but a reduction of the costs of ultra-pure silicon is still advantageous [2]. The material costs can be reduced through the use of thin-film solar cells, instead of relatively thick wafers. Crystalline (c-Si) has an indirect bandgap of 1.1 eV, resulting in a low optical absorption coefficient that causes weak absorption in near infrared (near-IR) region, and leading to an absorption length of 1 mm for a wavelength of 1100 nm. Light losses are most apparent from 750 to 1200 nm. In first-generation wafer based cells, the silicon has a surface texture (such as etched pyramids in wafer based c-Si) with a scale of around 10 µm to reduce reflection and trap light within the cell. This method is not applicable to thin film or second-generation solar cells, which may be only a few microns thick [3]. There is great scope for increased absorption using plasmonic and photonic effects to gain higher efficiencies and lower costs [4].

The concept of using white paint has been used to provide light trapping in thin-film solar cells and the basic theory of the optical behaviour has been first described in [5]. It has been shown previously that commercial white paint increases the short-circuit current density (*J_SC_*) and is a better back surface reflector than aluminium, and a transparent conducting oxide (TCO) and a detached aluminium mirror [6]. Commercial white paints use titania (TiO_2_) as the pigment, often rutile, which has a refractive index of 2.6. Benefits of using titania are that the material is non-toxic, cheap and widely available, stable to high temperatures and light resistant. However, in paint, the pigment titania (TiO_2_, often rutile with a refractive index of 2.6), is dispersed in an oil or latex based binder, with a refractive index of 1.4–1.7. Therefore, paint has the disadvantage of a relatively low refractive index contrast. It is well known that high index contrast is required to lead to strong photonic effects and higher reflection [7]. It has been demonstrated that a back reflector formed from high index nanoparticles without binder, can increase the performance of thin silicon devices (40% *J_SC_* increase for a 5-mm^2^ area device). The coating was formed using rutile particles of 270 nm in diameter, which were deposited in a strongly alkaline solution at pH 10 [8].

In the following, a novel coating method, *snow globe (SG) coating*, is presented that can be used to form an effective scattering back reflector for solar cells. The coating consists of coagulated high index particles of rutile (TiO_2_) and contains no binder, leading to a high refractive index contrast and very high reflectance. The coating shows better light trapping and enhances the cell performance more than two different commercial available paints when applied to a thin-film silicon solar cell. SG coating is executable in pH neutral media such as water and is therefore applicable to a wide range of solar cell types.

## 2. SNOW GLOBE COATING ON pc-Si THIN-FILM SOLAR CELL SHOWS BETTER CELL PERFORMANCE

### 2.1. Snow globe coating method

**Figure 1:**
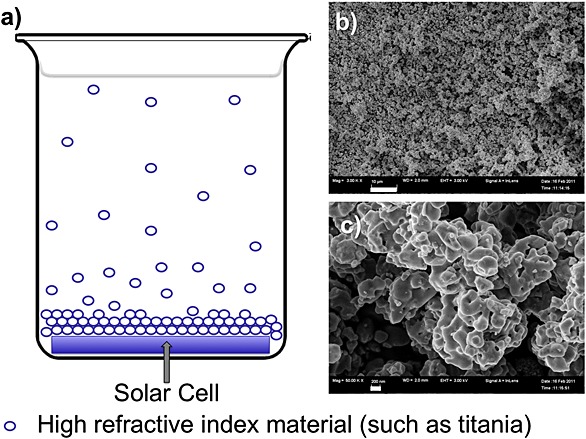
Snow globe (SG) coating method: (a) dispersed titania particles form a binder free coating (SG coating) after settling by gravity. Scanning electron micrograph of titania particles, (b) scale bar is 10 µm; (c) scale bar is 200 nm.

The SG coating method (see Figure 1) uses the fact that thick, uniform coatings of relatively large particles can be achieved by dispersion followed by settling by gravity, as in a children's snow globe. The technique allows large particles, which provide highly effective light scattering to be used. For the SG coating, titania particles without binder were used.

To form a uniform coating, the particles must first be dispersed in a liquid medium. Depending on the charge on the particles, they may either form a stable dispersion in the liquid, or may coagulate too fast, preventing the formation of a uniform coating. Dispersion is stable when the charge on the particles is sufficiently high that repulsive electrostatic forces exceed the attracting van der Waals forces. The surface charge can be tuned by adsorption of surfactants or ions, by varying the pH, or changing the concentration of ions.

**Figure 2:**
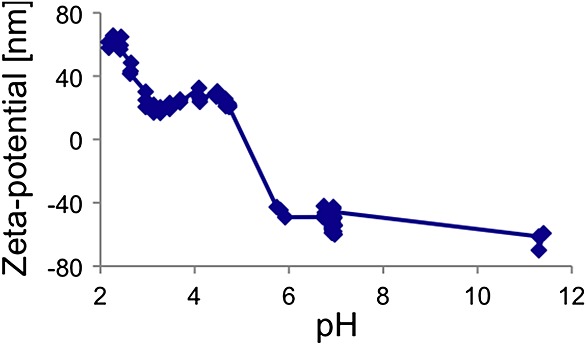
The pH as a function of the zeta-potential. The isoelectric point of titania is found at a pH of 5.3.

To choose a suitable medium in which the particles would form a stable dispersion, we determined the zeta potential of the titania particles, which is a measure of the charge of the particles. The zeta potential was obtained from measurements of the electrophoretic mobility of the charged particles as described in Ref. [9]. Figure 2 shows the results for the zeta potential as a function of pH of the medium. There is a high positive value of the zeta potential for strongly acidic solutions (pH < 3) and a high negative value of the zeta potential for neutral and alkaline solutions. These correspond to high charge on the titania particles and hence stable dispersions. In the pH regions 4–6, the zeta potential is relatively low, corresponding to low charge on the particles. Hence, solutions in this region would be expected to be unstable.

With the zeta potential result, either highly acidic, alkaline or neutral solutions could be used with SG coating. Acidic or alkaline solutions have the potential to harm the solar cell. Therefore, we chose to use a neutral solution of water to investigate the potential of SG coating for enhancing absorption in solar cells.

Particles (5 wt%) were dispersed using an ultrasonic bath (15 min) in 1000 ml of water (pH = 6–7) (The pH can be as low as 6 when tap water with dissolved carbon dioxide is used). The solar cell, a poly-crystalline silicon (pc-Si) 2 µm thin cell, was put in the bottom of a 2000 mL beaker and the suspension poured into the beaker. After the settling of the particles (about 2 h), the coated solar cell (SG coated) was (carefully) drawn out and dried. The coating is opaque and stable enough to be handled in a lab. The thickness is estimated to be about 0.2 mm. It can be easily turned over and is robust to small mechanical impacts. For commercial use, encapsulation is expected to be beneficial for the stability of the coating. SG coating is a process that can be upscaled. Titania is known to be dispersable in aqueous as well as non-aqueous (polar and non-polar) media #b[9]b[10]. Therefore, the proposed coating process could be extended to non-aqueous media as well, which may be beneficial for organic solar cells.

Solar cells with an area of 2 × 2 cm^2^ were used, consisting of an emitter layer, an absorber layer and a back surface field on a 3.3 mm thick Borofloat33 glass from Schott with a silicon nitride antireflection coating. The solar cells were formed using amorphous Si (a-Si) films deposited by e-beam deposition #b[11]b[12]. The a-Si is then crystallised with solid-phase crystallisation #b[11]b[13]. The Si films are 2 µm thick with 5% variation from centre to the edge #b[11]b[13]b[14].

The titania (TiO_2_) particles used in this project are provided by Treibacher Industrie AG (Treibach-Althofen, Austria) and have an average size of 1.106 µm. (TiO_2_ -100, L32090 Auftrag No. 4497). Particles of this size (similar in size to the wavelength of light) of metals or semiconductors should strongly interact with light [4]. X-ray diffraction measurements showed that the particles were rutile. Scanning electron micrographs of the material were taken using a ZEISS ultra plus (Extra high tension 3 kV, aperture 7.5 µm, working distance: 2.4 mm) and depicted in Figure 1(b) and (c).

### 2.2. Enhancement of external quantum efficiency

The spectral response of the solar cells was determined using a Xe lamp source, chopped at a frequency of 70 Hz and filtered by a monochromator over a bandwith of 300–1400 nm. The photocurrent at each wavelength, with a bandwidth of 10 nm, was measured with an SR570 preamplifier, and displayed as a voltage across an SR830 DSP lock-in amplifier. The external quantum efficiency (EQE) was then calculated from the known illumination intensity as the fraction of incident photons that are converted to electrical current. During the measurement, the beam is split so that half falls on the test cell and half on an internal reference cell with a known spectral response. Prior to the measurement, the instrument was calibrated. To avoid variation in the semiconductor material, the measurements were performed on the same spot of the solar cell. After performing SG coating, the cell was mounted on the instrument and measured. The coating was removed physically without moving the cell. The same spot then remeasured without the coating, providing the data for the plain cell. Then, the cell was painted *in situ* with commercial available paint and remeasured.

**Figure 3:**
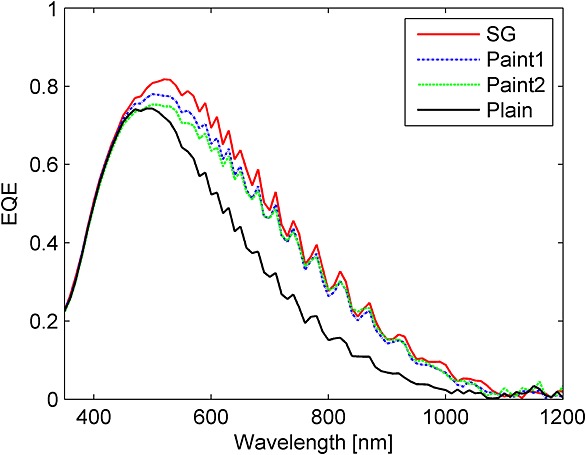
External quantum efficiency (EQE) of a plain (solid line), two painted (dotted line) for paint 1 and (dashed line) for paint 2 and a TiO_2_ coated cell (full line). The coating was performed with particles (about 1 μm) using the SG coating method.

The red, solid line in Figure 3 shows the enhancement of the EQE (number of electrons generated per number of incident photons) of a pc-Si thin-film solar cell coated by SG coating compared with two painted cases (blue, dotted line for paint 1 and green, dashed line for paint 2) and a plain (black, solid line) cell. The short circuit current *J_SC_* was calculated using Equation 1.


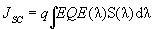
(1)

where *q* is the electron charge, *S* is the standard spectral photon density of sunlight at the earth's surface (Air Mass 1.5).

The snow globe coating results in a significant enhancement of 35% of *J_SC_*, compared with a planar cell. The commercially available paints provide an enhancement of 27% for paint 1 and 25% for paint 2 (Table I). The paint 1 used was ‘White Out’, also known as ‘Liquid paper’ or ‘Tipp-Ex’. Paint 2 was an acrylic paint called ‘Artists Titanium White’. In comparison, Ouyang *et al.* reported an enhancement of 28% when using contact paint with a very high reflectance (the cell used has a *J_SC_* of 14.5 mA/cm^2^ without back surface reflector) [15]. The coatings were optically characterised using a dual beam Perkin Elmer 1050 spectrophotometer, with an integrating sphere attachment to measure total reflection (R). The samples were measured with the light incident in the coating-silicon-glass direction to avoid absorption of light in the Si layer.

**Table I:** Enhancement of short circuit current *J_SC_* of titania coated solar cells

Sample	*J_SC_* (mA/cm^2^)	Enhancement (%)
Cell plain	13.9	–
Cell paint1	17.7	27
Cell paint2	17.4	25
Cell SG coated	18.7	35

**Figure 4:**
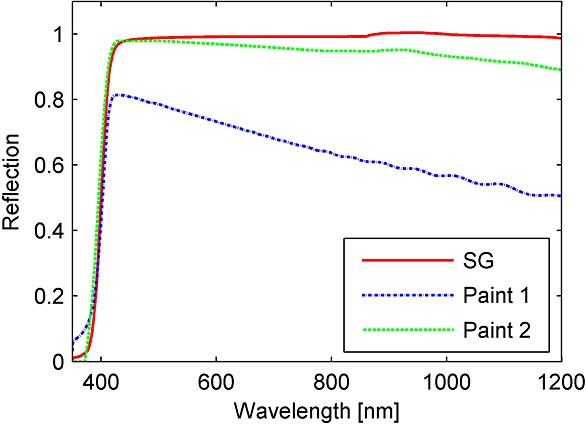
Experimental reflection of SG coating (solid line), paint 1 (dotted line) and paint 2 (dotted line).

The reflectance results are shown in Figure 4. SG coating has close to 100% reflectance at wavelengths above 400 nm. The novel coating is more reflective than both types of paint because of index contrast between the air and the TiO_2_ particles in the SG coating. The coating of paint 2 was opaque, so the loss through transmission is negligible, but there is some loss in reflectance probably attributable to absorption in the binder. For paint 1, absorption in the paint binder and transmission through the paint lower the reflection further.

## 3. MODELLING OF SNOW GLOBE COATING

A simple optical model was employed to investigate the origin of the enhanced light trapping efficiency of the SG coating compared with the different paints. The light trapping due to the rear-located diffuse scattering layers was modelled using the method of Goetzberger #b[16]b[17], with reflectance and the angular distribution of the scattered light as inputs [18]. The scattering distribution of the diffuse rear reflectors was modelled using a ‘narrowed Lambertian’ approach by applying the method of Cotter [19]. This method takes into account the refraction of the scattered light originating in a medium with *n*_eff_, as it enters the Si layer with *n*_Si_. The angular distribution is then given by: I _Θ_ = cos[asin(n_Si_/n_eff_ ∗ sin Θ)].

A transfer matrix method was used to calculate the reflection and transmission (and hence the absorption) of a layered stack consisting of a 2 µm Si layer coated with a 100 nm silicon nitride film on a semi-infinite glass superstrate. As in the experimental case, the light was incident from the glass superstrate. The *n*,*k* values for Si were taken from data from Keevers and Green [20], with *k* corrected for the higher absorption in pc-Si between 400 and 700 nm with data from He and Sproul [21], and the *n* value of the silicon nitride was taken as 2.0, which agrees well with experimentally determined values. The finite thickness of the glass was taken into account by assuming that the light that is within the escape cone for Si/glass but outside the escape cone for Si/air is returned to the silicon. Using this approach, the absorption in the silicon could be calculated for given values of *n*_*eff*_ and rear reflectance.

A wavelength-dependent ‘modelled internal quantum efficiency (IQE)’ was then defined by dividing the experimental EQE spectra for the plain cell by the calculated absorption of the plain cell (smoothed to extract the interference fringes).^1^ The calculated absorption in the Si with the different diffuse reflectors was multiplied by this modelled IQE to obtain a modelled EQE that could be directly compared with the experimental data.

The values of *n*_*eff*_ and the (wavelength independent) reflectance used in the model were chosen empirically by fitting the modelled data to the experimental EQE, using the measured reflectance as a starting point. It was found that this gave a better fit to the measured data than using the experimental reflectance as an input to the model. This was especially the case for paint 2, which has a lower reflectance than the other coatings. It is likely that the reflectance at an air/paint interface is lower than the internal reflectance at a Si/paint interface because in the latter, some of the light is totally internally reflected. This is consistent with the experimental *J_SC_* enhancements for paints 1 and 2, which are similar even though the reflectance of paint 1 is considerably higher.

**Figure 5:**
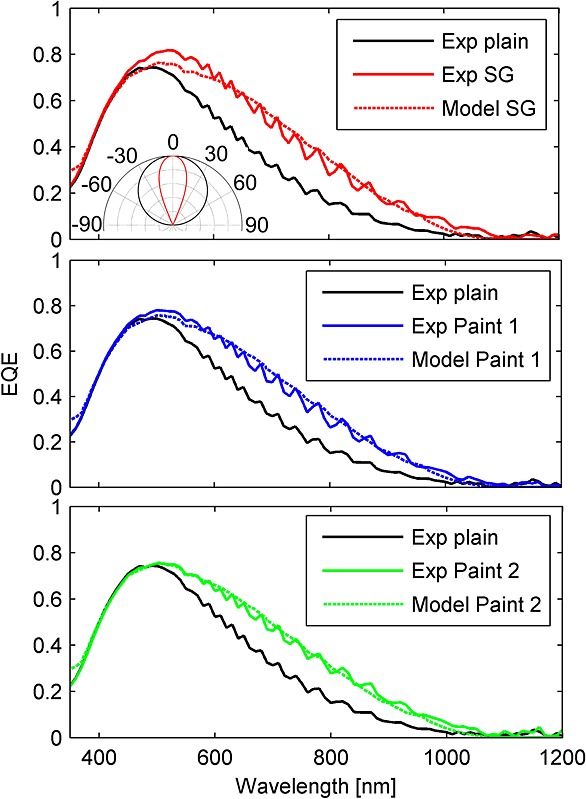
Enhancement of the experimental EQE compared with the modelled data for (top) SG, (middle) paint 1 and (bottom) paint 2 coatings. Inset: modelled narrowed Lambertian, I _Θ_ = cos[asin(n_Si_/n_eff_ ∗ sin Θ)], with *n*_*eff*_ = 1.4.

Figure 5 shows the modelled EQE spectra (dashed lines), compared with the experimentally measured data (solid lines). The modelled EQE spectra agree well with the experimental data for all three back reflectors. The inset in Figure 5 shows the angular distribution of the light scattered by the different coatings, all of which have an *n*_*eff*_ of 1.4. The inputs to the model and the resulting *J_SC_* enhancements (Λ) are summarised in Table II. It can be seen that the model also gives very good agreement with the experimental *J_SC_* enhancement.

**Table II:** Summary of enhancements for modelled and measured data for a 2-µm thick silicon film in the range 300–1200 nm

Coating	*n*_*eff*_	*R*	Λ_abs, MODEL_	Λ_abs, EXP_
SG coating	1.4	100	33	35
Paint 1	1.4	90	29	27
Paint 2	1.4	85	26	26
Lambertian	nSi	100	87	–

From these results, we can conclude that the advantage of the SG coating is its very high reflectance. This is due to the strong scattering provided by the high refractive index contrast between the rutile TiO_2_ particles and air, and the lack of a binder that could lead to parasitic absorption. The *n*_*eff*_ of the coating is still relatively low however, meaning the angular distribution of the scattered light is narrowed in the Si layer. Optimising particle size may result in a broader angular range and lead to larger enhancements. The paints show more modest light trapping efficiencies because of the presence of a binder, which reduces scattering and leads to absorption.

For the SG coating, the model underestimates the enhancement in the EQE at wavelengths below 650 nm. The underestimation in the model was preserved when an ideal Lambertian type reflector was applied, suggesting that it is not due to an optical effect. Similar anomalous EQE enhancements have been seen at short wavelengths by Lee [8]. We attribute the anomalously high EQE enhancements at short wavelengths to an increase in the IQE of the cells attributable to a redistribution of generated charge carriers: when a strong rear reflector is applied on the field distribution, and hence the generated carrier distribution inside the Si is changed, and carriers are no longer generated preferentially at the surface of the cell. As the Si interface is a region of high recombination, this reduces the number of carriers lost before collection at the contacts, and hence increases the IQE.

## 4. CONCLUSION

We have demonstrated a novel method, SG coating, which gives enhanced EQE performance in thin-film silicon solar cells. It is formed by rutile particles and, unlike paint, contains no binder, resulting in higher refractive index contrast of the coating. It shows higher reflectivity at wavelengths above 400 nm than both investigated paint cases. As the reflectance is lower, but the angular spectrum is wider for paint, we can conclude that the most important factor is the reflectance, which is likely to be lower for paint because of absorption in the binder.

The coating can be formed using fairly large high-index material particles in mild, pH neutral, conditions and is applicable to many different kinds of solar cells. Furthermore, it does not lead to an increase of surface recombination, which occurs with other light trapping techniques [4]. Another major advantage is that the method is compatible to other light trapping techniques such as plasmonics and surface textures that could benefit from improved reflection 22.

## Footnotes

1 The modelled IQE was fit with a quadratic at long wavelengths (above 850 nm) to avoid unphysical values over 100% caused by noise in the experimental data as the calculated absorption tends to zero.

## References

[1] Ginley D, Green MA, Collins R (2008). Solar energy conversion toward 1 terawatt. MRS Bulletin.

[2] Wenham SR, Green MA, Watt ME, Corkish R (2007). Applied Photovoltaics, Earthscan.

[3] Green M (2007). Thin-film solar cells: review of materials, technologies and commercial status. Journal of Materials Science: Materials in Electronics.

[4] Catchpole KR, Mokkapati S, Beck FJ, Wang EC, McKinley A, Basch A, Lee J (2011). Plasmonics and nanophotonics for photovoltaics. MRS Bulletin.

[5] Cotter JE, Hall RB, Mauk MG, Barnett AM (1999). Light trapping in silicon-film solar cells with rear pigmented dielectric reflectors. Progress in Photovoltaics: Research and Applications.

[6] Berger O, Inns D, Aberle AG (2007). Commercial white paint as back surface reflector for thin-film solar cells. Solar Energy Materials & Solar Cells.

[7] Reufer M, Rojas-Ochoa LF, Eiden S, Sáenz JJ, Scheffold F (2007). Transport of light in amorphous photonic materials. AIP.

[8] Lee BG, Stradins P, Young DL, Alberi K, Chuang T-K, Couillard JG, Branz HM (2011). Light trapping by a dielectric nanoparticle back reflector in film silicon solar cells. Applied Physics Letters.

[9] Taloyan AM, Bankiewicz DS (2012). Coagulation: Kinetics, Structure Formation and Disorders.

[10] Basch A, Strnad S, Ribitsch V (2009). Substrate-induced coagulation (SIC) of nano-disperse titania in non-aqueous media: the dispersibility and stability of titania in *N*-methyl-2-pyrrolidone. Colloids and Surfaces A: Physicochemical and Engineering Aspects.

[11] Kunz O, Ouyang Z, Varlamov S, Aberle AG (2009). 5% efficient evaporated solid-phase crystallised polycrystalline silicon thin-film solar cells. Progress in Photovoltaics: Research and Applications.

[12] Green MA, Basore PA, Chang N, Clugston D, Egan R, Evans R, Hogg D, Jarnason S, Keevers M, Lasswell P, O'Sullivan J, Schubert U, Turner A, Wenham SR, Young T (2004). Crystalline silicon on glass (CSG) thin-film solar cell modules. Solar Energy.

[13] Kunz O, Ouyang Z, Wong J, Aberle AG (2008). Device fabrication scheme for evaporated SPC poly-Si thin film solar cells on glass (EVA). IUMRS-ICEM, International Conference on Electronic Materials.

[14] Söderström T, Wang Q, Omaki K, Kunz O, Ong D, Varlamov S (2011). Light confinement in e-beam evaporated thin film polycrystalline silicon solar cells. Physica status solidi (RRL) - Rapid Research Letters.

[15] Ouyang Z, Pillai S, Beck F, Kunz O, Varlamov S, Catchpole KR, Campbell P, Green MA (2010). Effective light trapping in polycrystalline silicon thin-film solar cells by means of rear localized surface plasmons. Aplied Physics Letters.

[16] Götzberger A

[17] Green MA (2002). Lambertian light trapping in textured solar cells and light-emitting diodes: analytical solutions. Progress in Photovoltaics: Research and Applications.

[18] Beck FJ, Mokkapati S, Catchpole KR (2011). Light trapping with plasmonic particles: beyond the dipole model. Optics Express.

[19] Cotter JE (1998). Optical intensity of light in layers of silicon with rear diffuse reflectors. Journal of Applied Physics.

[20] Keevers MJ, Green MA (1995). Absorption edge of silicon from solar cell spectral response measurements. Applied Physics Letters.

[21] He S, Sproul AB (2010). Optical properties of evaporated poly-Si thin-films on glass. Thin Solid Films.

[22] Basch A, Beck FJ, Söderström T, Varlamov S, Catchpole KR

